# Elliptical and spherical heads show similar obligate glenohumeral translation during axial rotation in total shoulder arthroplasty

**DOI:** 10.1186/s12891-023-06273-5

**Published:** 2023-03-07

**Authors:** Lukas N. Muench, Matthew Murphey, Bridget Oei, Cameron Kia, Elifho Obopilwe, Mark P. Cote, Augustus D. Mazzocca, Daniel P. Berthold

**Affiliations:** 1 Department of Orthopaedic Sports Medicine, Klinikum rechts der Isar, Technical University of Munich, Munich, Germany; 2 Department of Orthopaedic Surgery, UConn Health, Farmington, CT USA; 3 Massachusetts General Brigham, Massachusetts General Hospital, Harvard Medical School, Boston, MA USA; 4 Department of Orthopaedics and Trauma Surgery, Musculoskeletal University Center Munich (MUM), University Hospital, LMU Munich, Munich, Germany

**Keywords:** Humeral head, Elliptical, Spherical, Prosthesis design, Total shoulder arthroplasty

## Abstract

**Background:**

Elliptical shape humeral head prostheses have been recently proposed to reflect a more anatomic shoulder replacement. However, its effect on obligate glenohumeral translation during axial rotation compared to a standard spherical head is still not well understood. The purpose of the study was to compare obligate humeral translation during axial rotation using spherical and elliptical shaped humeral head prostheses. It was hypothesized that the spherical head design would show significantly more obligate translation when compared to the elliptical design.

**Methods:**

Six fresh-frozen cadaveric shoulders were utilized for biomechanical testing of internal (IR) and external (ER) rotation at various levels of abduction (0°, 30°, 45°, 60°) with lines of pull along each of the rotator cuff muscles. Each specimen underwent the following three conditions: (1) native; total shoulder arthroplasty (TSA) using (2) an elliptical and (3) spherical humeral head implant. Obligate translation during IR and ER was quantified using a 3-dimensional digitizer. The radius of curvature of the superoinferior and anteroposterior dimensions of the implants was calculated across each condition.

**Results:**

Posterior and inferior translation as well as compound motion of spherical and elliptical heads during ER was similar at all abduction angles (P > 0.05, respectively). Compared to the native humeral head, both implants demonstrated significantly decreased posterior translation at 45° (elliptical: P = 0.003; spherical: P = 0.004) and 60° of abduction (elliptical: P < 0.001; spherical: P < 0.001). During internal rotation at 0° abduction, the spherical head showed significantly more compound motion (P = 0.042) compared to the elliptical head. The spherical implant also demonstrated increased anterior translation and compound motion during internal rotation at 60° abduction (P < 0.001) compared to the resting state. This difference was not significant for the native or elliptical head design at this angle (P > 0.05).

**Conclusion:**

In the setting of TSA, elliptical and spherical head implants showed similar obligate translation and overall compound motion during axial rotation. A gained understanding of the consequences of implant head shape in TSA may guide future surgical implant choice for better recreation of native shoulder kinematics and potentially improved patient outcomes.

**Level of evidence:**

Controlled Laboratory Study.

## Background

Unlike other ball and socket joints, the glenohumeral articulation differs in that the glenoid cavity is too shallow to capture the entire humerus during motion [1]. As such, the humeral head undergoes obligate translation at extremes of motions in order to remain centered within the glenoid cavity [1, 2]. According to Harryman et al., glenohumeral translation is considered obligate in that it cannot be prevented by application of an oppositely directed force of 30 to 40 N [1]. Further, increased obligate translation can be a result of an asymmetrically tight capsule [1]. While this stability is often provided by soft tissue restraints, [3] recreation of obligate translation during anatomic total shoulder arthroplasty (TSA) may rely greater on implant design in order to recreate the natural interaction of the glenohumeral joint [4].

Improper glenohumeral translation after TSA may have important implications with regard to implant longevity and patient outcomes [5, 6]. As the shoulder moves to near full extension and external rotation, the native humerus will translate posteriorly in order to remain centered in the glenoid, and similarly will translate anteriorly in cross-body position [1, 2]. Interestingly, Karduna et al. compared translation before and after anatomic arthroplasty, finding that active translation had greater dependence on the articular conformity rather than capsular ligaments [4]. This has led to a greater importance on recreating the anatomic relationship of the humeral joint through the implant interface.

While once thought to be perfectly spherical in shape, several recent anatomic studies of the shoulder have differed from the previous notion that the humeral head is a perfect sphere [7–11]. Previous biomechanical studies have shown that a non-spherical prosthetic head more accurately replicated native glenohumeral joint properties in terms of kinematics, translation, and rotational range of motion [12–14]. More importantly, the non-spherical humeral head design resulted in increased glenohumeral translation during humeral axial rotation along with increased micromotion of the glenoid component when compared to the spherical head design [13, 15]. However, these studies were limited to the prior resection of rotator cuff muscles and capsuloligamentous structures, leaving the effect of these soft tissue restraints on glenohumeral translation unknown. As the spherical design is characterized by a greater volume in the anteroposterior dimension compared to the elliptical head, tightening of the anterior part (during external rotation) or posterior part (during internal rotation) of the capsule may push the spherical head more posteriorly or anteriorly during axial rotation. This may especially be observed at higher abduction angles with the glenohumeral joint being more constraint.

Although long-term clinical implications have not been shown, understanding the actual translation that occurs could improve further implant design [16]. Thus, the purpose of the present study was to compare obligate humeral translation during axial rotation using spherical and elliptical shaped humeral head prostheses. The authors hypothesized that the spherical head design would show significantly more obligate translation when compared to the elliptical design.

## Methods

Six fresh-frozen, cadaveric shoulders with a mean age of 62.7 ± 9.2 years (range 48–74 years) were used for the study (Science Care Inc., Phoenix, AZ, USA). All specimens underwent visual and radiographic inspection to exclude those with tears of the rotator cuff tendons and capsule, moderate to severe osteoarthritis, bony defects, or joint contractures. As de-identified specimens were not considered to constitute human subjects research, prior Institutional Review Board approval was not required.

### Specimen preparation

After having been thawed overnight at room temperature, specimens were dissected free of skin, subcutaneous tissue, and muscles. Rotator cuff muscles, capsule, and the coracoacromial ligament were carefully preserved. Under fluoroscopy control (Mini C-Arm, GE Medical Systems Inc.) a 2.0 mm K-wire was drilled parallel to the glenoid surface from posterior to anterior at the middle of the superior–inferior diameter. A second 2.0 mm k-wire was drilled from inferior to superior parallel to the glenoid. The scapulae were trimmed using an oscillating saw and potted in a custom rectangular box with the glenoid surface being aligned parallel to the floor. After being shortened, the humerus was centered and potted in a poly-vinyl chloride pipe (PVC; diameter, 3.8 cm; length, 7 cm) using bone cement, leaving only 2 cm of the proximal humeral shaft exposed, in order to minimize diaphyseal bending moments [17, 18].

### Testing setup

**Fig. 1:**
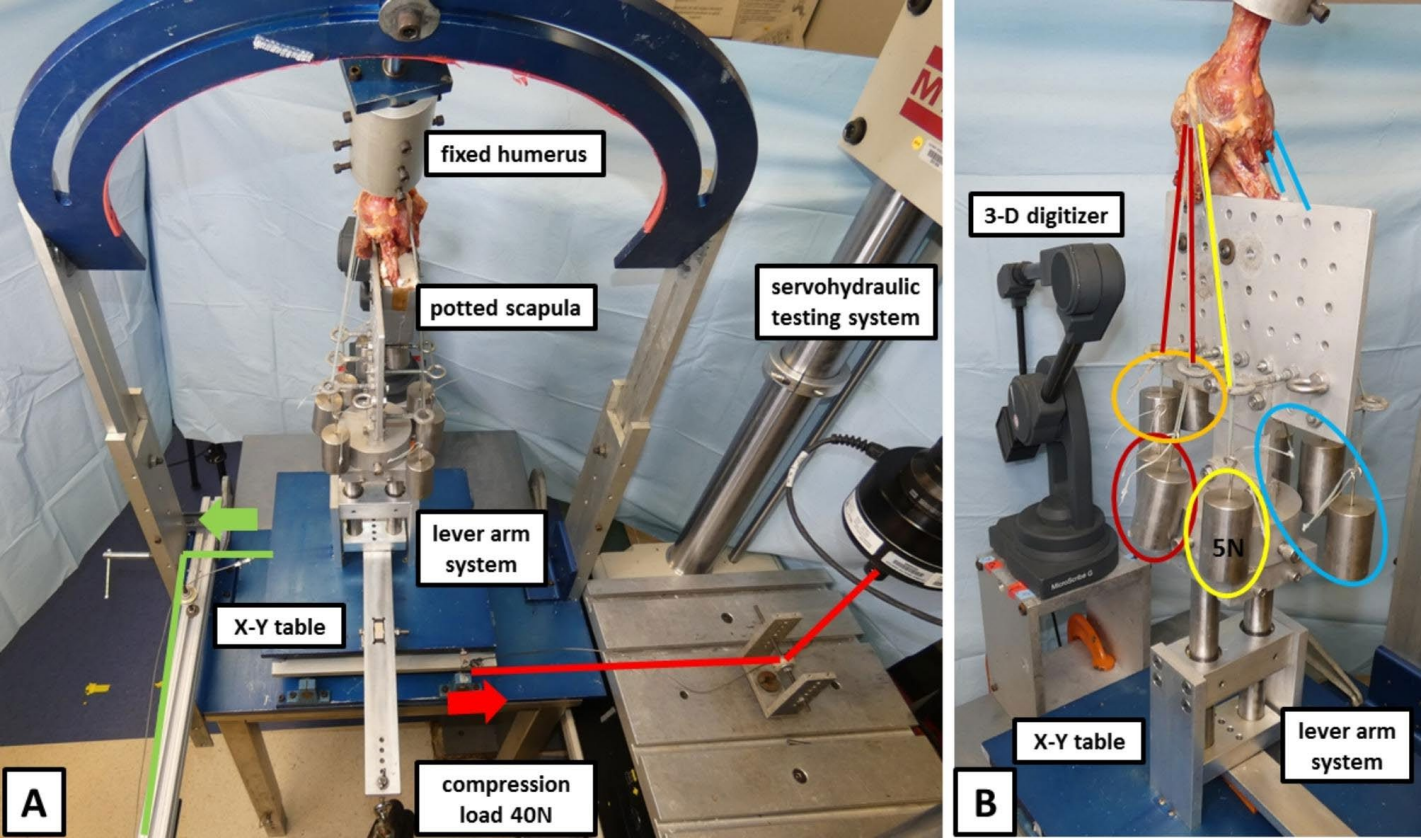
***(A)*** Displaying a right shoulder specimen mounted to the shoulder testing rig. The scapula is fixed to a vertical linear bearing translator and lever arm system on top of an X-Y table, allowing for glenohumeral translation in the anteroposterior and superoinferior direction. During testing, an axial compression load of 40 N is constantly applied via the lever arm of the X-Y table to center the joint. As the humerus is fixed in the testing rig, the oppositely directed force of 30 N is consequently applied to the X-Y table (glenoid) in the posterior direction during external rotation (green arrow) and in the anterior direction (red arrow) during internal rotation. The force is applied via a friction-less cable, which is attached to a servohydraulic testing system or 30 N hanging weight, depending on the direction of force. ***(B)*** The rotator cuff muscles are loaded based on physiological cross-sectional area ratios with multiple lines of pull. Specifically, two lines of pull are used for the supraspinatus (orange), three for the subscapularis (blue), two for the infraspinatus (red), and one for the teres minor (yellow). Each line of pull is loaded with 5 N, resulting in a total load of 40 N

The specimens were mounted to a validated shoulder testing rig as previously described, which allowed for positioning of the glenohumeral joint in 6 degrees of freedom (Fig. 1A) [18–22]. With the glenoid surface being in a horizontal position parallel to the floor, the scapula was fixed to a vertical linear bearing translator and lever arm system on top of an X-Y table, allowing for glenohumeral translation in the anteroposterior and superoinferior direction. The rotation of the humerus was defined as neutral with the bicipital groove being aligned with the anterior margin of the acromion according to Selecky et al. [17, 23]. The rotator cuff muscles were loaded based on physiological cross-sectional area ratios with multiple lines of pull as previously described [24, 25]. Specifically, two lines of pull were used for the supraspinatus, three for the subscapularis, two for the infraspinatus, and one for the teres minor (Fig. 1B) [18, 25]. Each line of pull was loaded with 5 N, resulting in a total load of 40 N [18, 25].

### Biomechanical Testing

During testing, an axial compression load of 40 N was constantly applied via the lever arm of the X-Y table to center the joint [13]. Each specimen underwent the three following conditions: (1) native, TSA with a (2) matched-fit elliptical head and (3) matched-fit spherical head. According to Jun et al., [13] 50° of internal and 50° of external axial rotation were alternatingly applied to the humerus at 0°, 30°, 45° and 60° of glenohumeral abduction in the scapular plane.

According to Harryman et al., [1] who stated that glenohumeral translation is considered obligate in that it cannot be prevented by application of an oppositely directed force of 30 N, an anterior directed force was applied to the humeral head during external rotation due to the posterior translation of the humeral head. Conversely, a posterior directed force was applied to the humeral head during internal rotation due to the anterior translation of the humeral head [1]. As the humerus was fixed in the testing rig, the force of 30 N was consequently had to be applied to the X-Y table (glenoid) in the posterior direction during external rotation and in the anterior direction during internal rotation. The force was applied via a friction-less cable, which was attached to a servohydraulic testing system (Mini Bionix 858; MTS) or 30 N hanging weight, depending on the direction of force (Fig. 1A).

By means of a 3-dimensional digitizer (MicroScribe G2; Immersion) with a position accuracy of 0.23 mm, the position of the X-Y table was measured by carefully digitizing the center of a defined groove on the X-Y table without relevant influence by touching off with the digitizer. The position of the groove was determined at the beginning (start position) and the end (end position) of each application of internal and external rotation. Changes in the position represented the glenohumeral translation and were given in anteroposterior (x-axis) and superoinferior (y-axis) directions. In addition, overall compound motion during internal and external rotation was calculated as the square root of the sum of the squared anteroposterior (x-axis) and squared superoinferior (y-axis) translation.

Internal and external rotation were alternatingly applied five times for every condition. Values of each specimen were then averaged and presented as the final values. Throughout entire testing, specimens were not removed from the testing rig, nor was the testing rig disassembled. The protruding spike of the trunnion allowed for easily switching between the elliptical and spherical head design during testing. To avoid selection bias, the order of glenohumeral abduction positions (0°, 30°, 45°, 60°) and head designs (elliptical or spherical) were randomly assigned.

### Radius of curvature

**Fig. 2:**
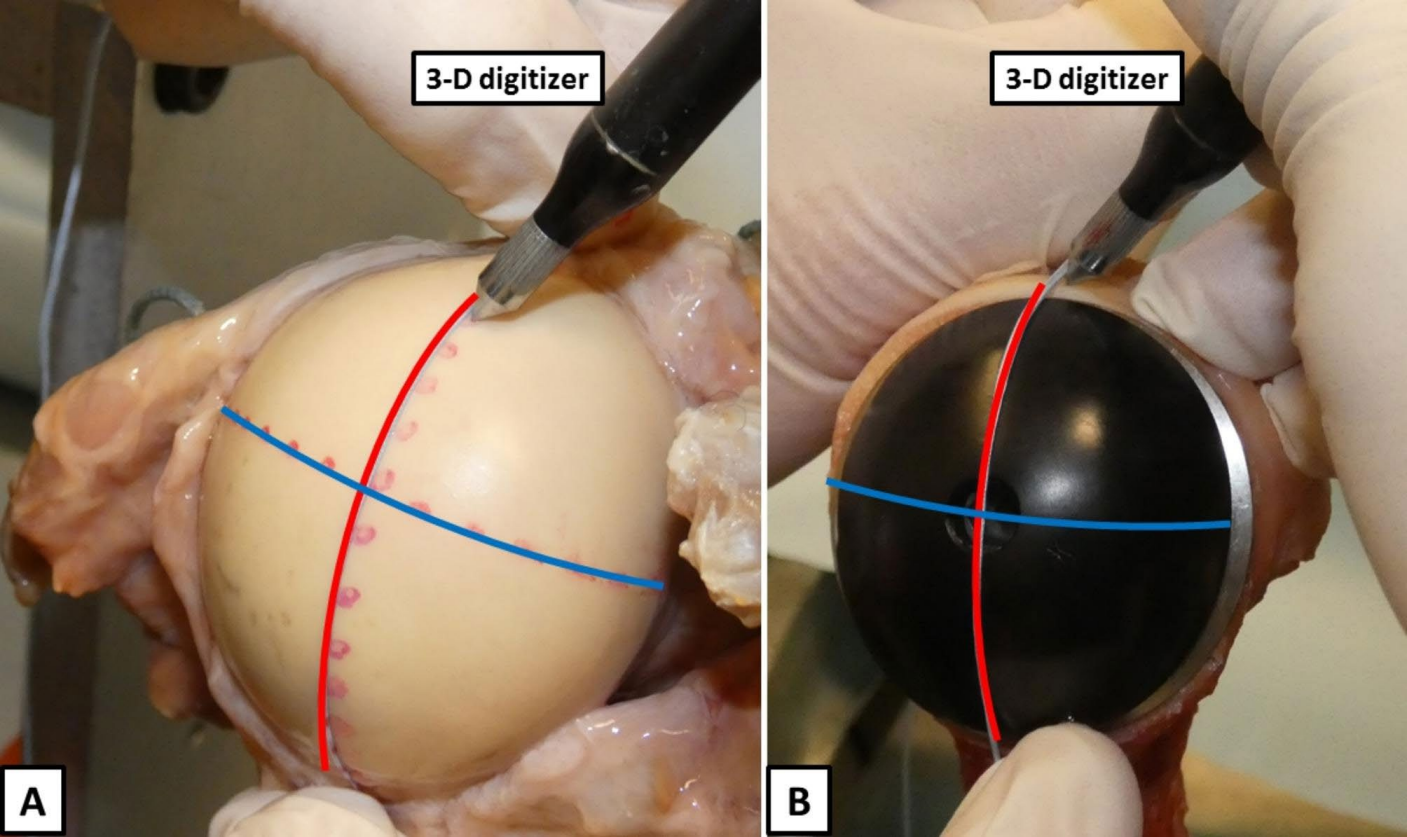
***(A)*** Prior to testing, the superoinferior (red) and anteroposterior (blue) dimension of the native humeral head is carefully digitized in 0.5 mm intervals by using the 3-dimensional digitizer. ***(B)*** Following completion of translational testing, each specimen is disarticulated, and all remaining soft tissue is resected. The matched-fit elliptical is placed on the trunnion and then digitized as previously described. The data is implemented in a 3D computer graphics software and the radius of curvature of the superoinferior and anteroposterior dimensions are calculated

Prior to testing, the superoinferior and anteroposterior dimension of the native humeral head was carefully digitized in 0.5 mm intervals by using the 3-dimensional digitizer (MicroScribe G2; Immersion) (Fig. 2A). Following completion of translational testing, each specimen was disarticulated, and all remaining soft tissue was resected. The matched-fit elliptical (Fig. 2B) and spherical head were each placed on the trunnion and then digitized as previously described. The data was implemented in a 3D computer graphics software (Rhinoceros 3D, McNeel, Boston, USA) and the radius of curvature of the superoinferior and anteroposterior dimensions were calculated for each condition.

### Surgical technique

Total shoulder arthroplasty was performed using an anatomic stemless implant (Eclipse system, Arthrex Inc., Naples, FL, USA) according to a previously described technique [26, 27]. Each surgery was performed by the same surgeon (L.N.M.) in order to minimize performance bias. Oriented along the specimen’s anatomic retro-torsion, two 1.6 mm K-wires were pre-drilled in line with the desired resection plane, exiting the opposite cortex at the boundary of the articular cartilage. Guided by the two K-wires, an osteotomy was performed using an oscillating saw. After measuring the anterior-posterior dimension of the resected humeral head, the size of the baseplate (trunnion) was determined. The trunnion was then fixed to the resected humeral neck and a hollow screw was inserted. The custom-made trunnion used for this study was additionally secured with a small, protruding spike, to allow for easily switching the different prosthetic heads during testing.

Glenoid replacement was performed using a keeled glenoid system (Univers II, Arthrex Inc., Naples, FL, USA). A glenoid guide was placed on the central axis of the exposed articular surface of the glenoid, with the guide handle being oriented in line with the anatomic slope of the anterior neck. Following preparation, a keeled glenoid implant was inserted in the created slot and impacted.

### Humeral head prosthetic design

Both elliptical and spherical prosthetic humeral heads were custom made (Arthrex Inc., Naples, FL, USA). The designs, including equations for dimension width, radius of curvature, and height, were chosen according to previously published studies [7, 8]. A small hole in the undersurface allowed for securely placing the humeral head prosthesis on the protruding spike of the trunnion, avoiding rotation of the head prosthesis during testing and allowing for easily switching heads between testing conditions.

### Statistical analysis

A power analysis was carried out to determine detectable differences in translation, using standard deviations estimated from the literature as well as pilot data from our laboratory [12]. Assuming a common standard deviation of 1.5 mm, a sample size of 6 specimens would provide 80% power to detect a 2.5 mm difference in translation at an α level of 0.05. Descriptive statistics including mean and standard deviation (SD) as well as median and interquartile range (IQR) were calculated to characterize the groups. Differences in translation between implants were assessed using multilevel mixed effects generalized linear models. A random intercept was used to account for specimens in different conditions. For each analysis, the distribution of the residual was examined and found to conform to a normal distribution. Comparisons of marginal mean values were carried out with adjustment for multiple comparisons using the Holm-Bonferroni sequential correction method, in case of initial statistical significance. A p value of 0.05 was set to be statistically significant. All statistical analyses were conducted using Stata 15 software (StataCorp. 2017. Stata Statistical Software: Release 15. College Station, TX: StataCorp LLC).

## Results

### Obligate Glenohumeral translation

**Fig. 3:**
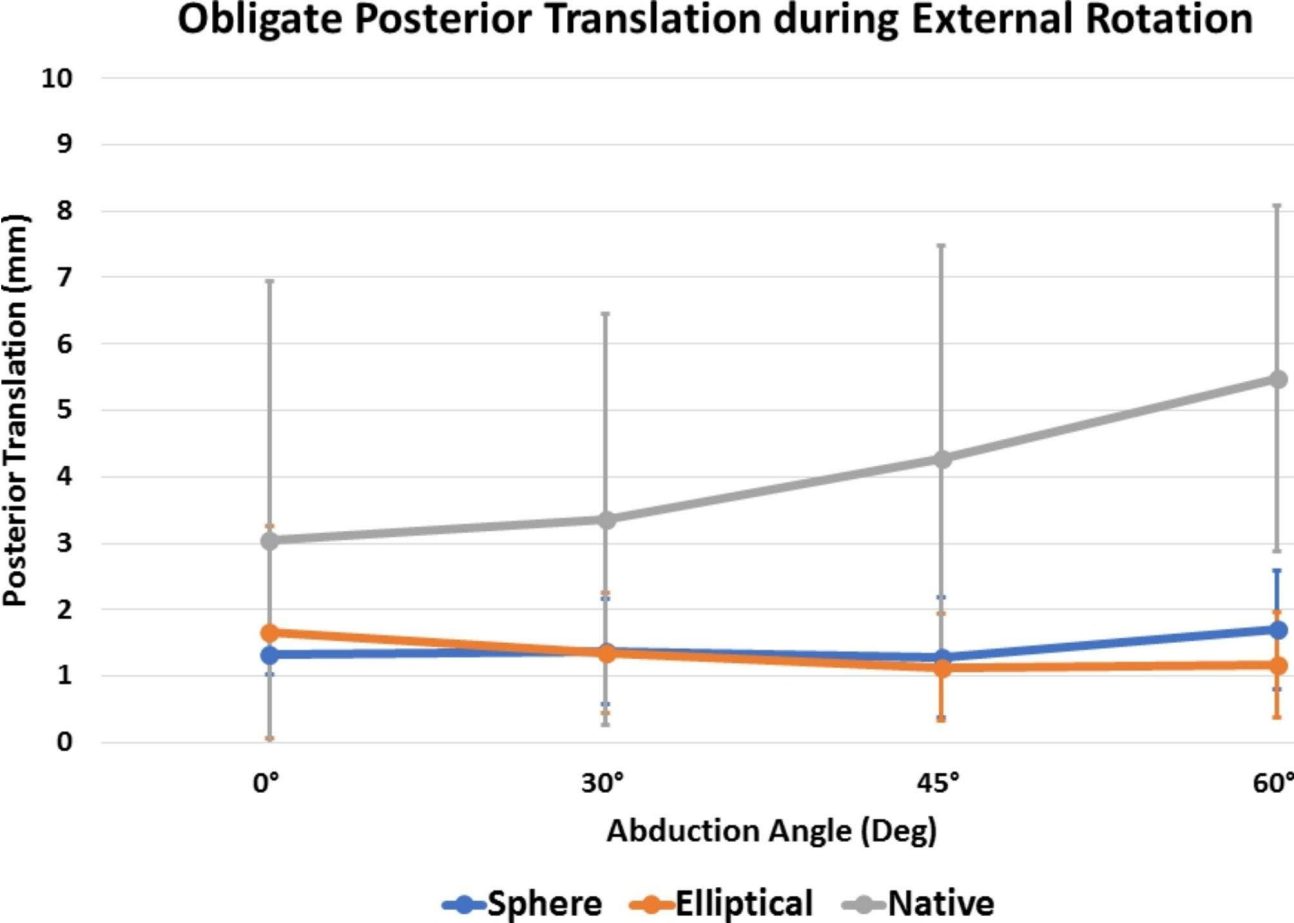
Obligate posterior translation (mm) during external rotation

**Fig. 4:**
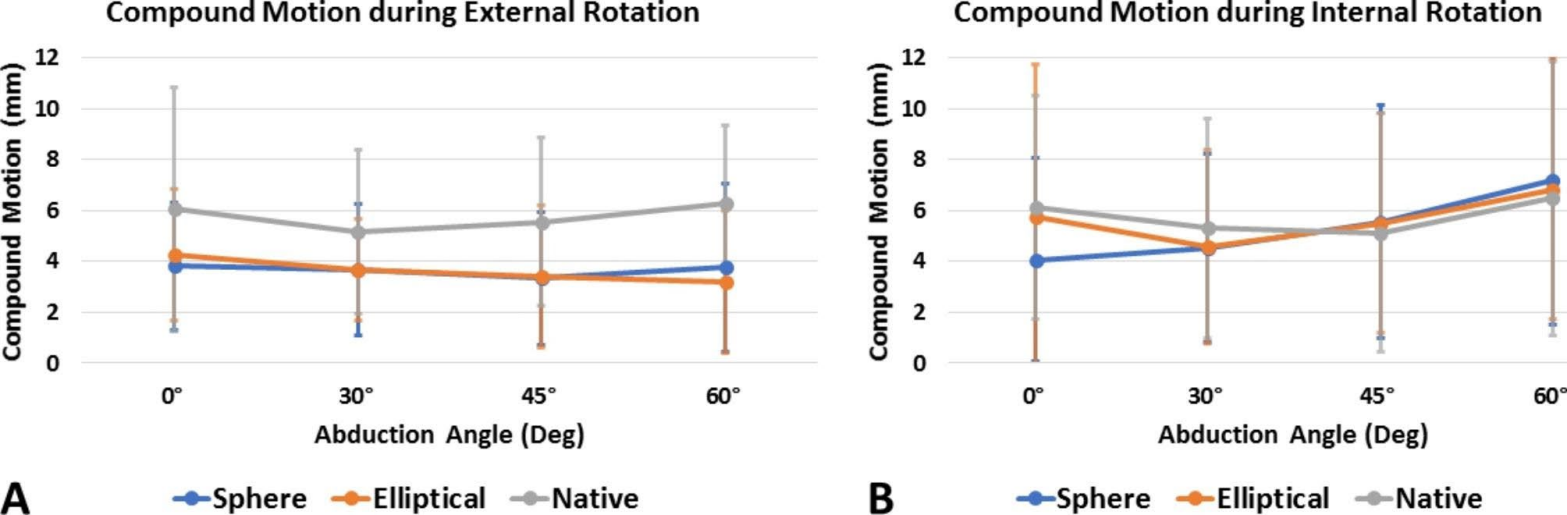
Overall compound motion (mm) during external ***(A)*** and internal ***(B)*** rotation

Elliptical and spherical heads showed similar obligate posterior translation (Table 1; Fig. 3), inferior translation (Table 2), and overall compound motion (Table 3; Fig. 4A) during external rotation at all tested abduction angles (P > 0.05, respectively). For both implant groups, obligate posterior translation during external rotation was decreased compared to the native state at all angles, however, this did only reach statistical significance at 45° (elliptical vs. native: P = 0.003; spherical vs. native: P = 0.004) and 60° (elliptical vs. native: P < 0.001; spherical vs. native: P < 0.001). In addition, the native humeral head showed significantly more obligate posterior translation (P = 0.007) and inferior translation (P < 0.001) at 60° of abduction during external rotation when compared to the resting position, whereas this was not observed for either the elliptical or spherical head design (P > 0.05, respectively).

**Fig. 5:**
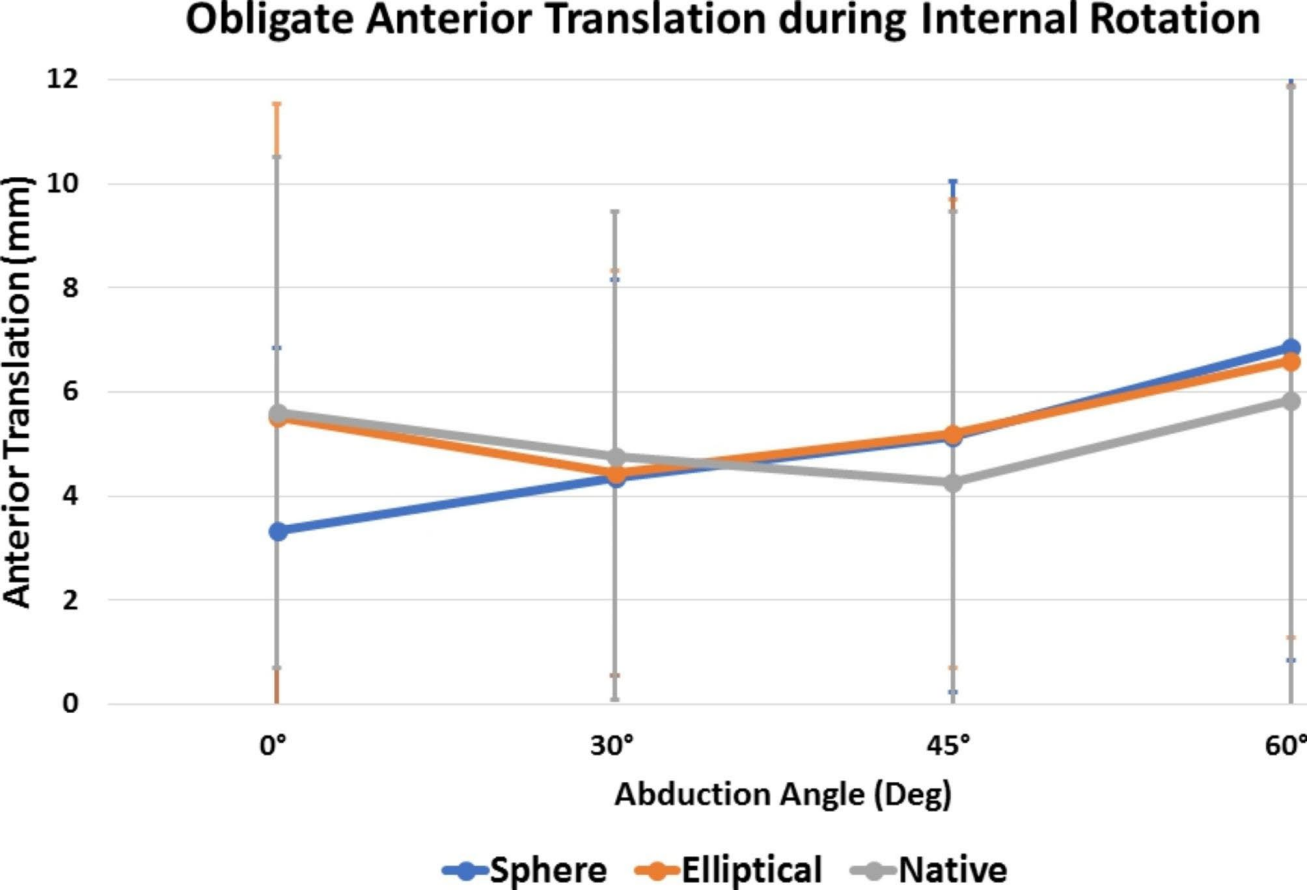
Obligate anterior translation (mm) during internal rotation

During internal rotation, spherical heads showed significantly more obligate anterior translation (P = 0.024; Fig. 5) and compound motion (P = 0.042; Fig. 4B) in the resting position when compared to elliptical heads. For both implant groups, obligate anterior translation during internal rotation was increased compared to the native state at higher abduction angles, however, this did not reach statistical significance (P > 0.05, respectively). In addition, the spherical design showed significantly more obligate anterior translation (P < 0.001) and compound motion (P < 0.001) at 60° of abduction during internal rotation when compared to the resting position, whereas this was not observed for either the native humeral head or the elliptical head design (P > 0.05, respectively).

**Table 1:** Obligate posterior glenohumeral translation (mm) during external rotation (ER) and obligate anterior glenohumeral translation (mm) during internal rotation (IR). Abbreviations: IQR = interquartile range; SD = standard deviation

			Posterior Translation (mm) during ER	Anterior Translation (mm) during IR
**0°**	**Native**	*mean ± SD*	3.0 ± 3.9	5.6 ± 4.9
		*median*	1.8	4.3
		*IQR*	1.5	5.4
	**Elliptical**	*mean ± SD*	1.7 ± 1.6	5.5 ± 6.0
		*median*	1.4	2.4
		*IQR*	0.8	8.6
	**Sphere**	*mean ± SD*	1.3 ± 0.3	3.3 ± 3.5
		*median*	1.3	1.8
		*IQR*	0.5	4.2
**30°**	**Native**	*mean ± SD*	3.4 ± 3.1	4.8 ± 4.7
		*median*	2.6	3.5
		*IQR*	4.9	8.9
	**Elliptical**	*mean ± SD*	1.4 ± 0.9	4.4 ± 3.9
		*median*	1.3	2.9
		*IQR*	0.6	6.9
	**Sphere**	*mean ± SD*	1.4 ± 0.8	4.4 ± 3.8
		*median*	1.5	3.1
		*IQR*	1.3	6.1
**45°**	**Native**	*mean ± SD*	4.3 ± 3.2	4.3 ± 5.2
		*median*	4.5	3.9
		*IQR*	2.1	4.0
	**Elliptical**	*mean ± SD*	1.1 ± 0.8	5.2 ± 4.5
		*median*	0.7	3.8
		*IQR*	1.4	7.4
	**Sphere**	*mean ± SD*	1.3 ± 0.9	5.1 ± 4.9
		*median*	1.2	3.4
		*IQR*	1.8	9.6
**60°**	**Native**	*mean ± SD*	5.5 ± 2.6	5.8 ± 6.0
		*median*	6.4	3.4
		*IQR*	1.6	11.0
	**Elliptical**	*mean ± SD*	1.2 ± 0.8	6.6 ± 5.3
		*median*	1.3	4.6
		*IQR*	1.3	9.1
	**Sphere**	*mean ± SD*	1.7 ± 0.9	6.8 ± 6.0
		*median*	1.6	4.9
		*IQR*	1.2	11.2

**Table 2:** Inferior glenohumeral translation during external (ER) and internal (IR) rotation. Abbreviations: IQR = interquartile range; SD = standard deviation

			Translation (mm) during ER	Translation (mm) during IR
**0°**	**Native**	*mean ± SD*	4.6 ± 3.7	0.8 ± 1.2
		*median*	4.4	0.4
		*IQR*	6.4	0.8
	**Elliptical**	*mean ± SD*	3.0 ± 3.4	0.5 ± 1.4
		*median*	2.4	0.2
		*IQR*	4.0	0.8
	**Sphere**	*mean ± SD*	3.2 ± 2.9	0.5 ± 1.0
		*median*	2.5	0.4
		*IQR*	3.4	0.4
**30°**	**Native**	*mean ± SD*	3.0 ± 2.8	0.7 ± 1.2
		*median*	2.3	0.5
		*IQR*	2.1	0.8
	**Elliptical**	*mean ± SD*	2.8 ± 2.6	0.3 ± 0.6
		*median*	2.7	0.3
		*IQR*	3.4	0.6
	**Sphere**	*mean ± SD*	3.0 ± 3.0	0.5 ± 0.7
		*median*	2.2	0.5
		*IQR*	3.4	1.2
**45°**	**Native**	*mean ± SD*	2.6 ± 2.8	1.1 ± 1.7
		*median*	1.5	0.3
		*IQR*	0.9	0.7
	**Elliptical**	*mean ± SD*	2.7 ± 3.2	0.8 ± 1.0
		*median*	1.5	0.7
		*IQR*	5.7	1.3
	**Sphere**	*mean ± SD*	2.6 ± 3.0	0.9 ± 1.1
		*median*	1.7	0.9
		*IQR*	3.9	2.0
**60°**	**Native**	*mean ± SD*	2.4 ± 2.7	0.8 ± 1.5
		*median*	1.7	0.1
		*IQR*	2.8	2.0
	**Elliptical**	*mean ± SD*	2.5 ± 3.2	0.9 ± 1.0
		*median*	1.5	0.7
		*IQR*	3.5	1.6
	**Sphere**	*mean ± SD*	2.9 ± 3.6	1.0 ± 1.2
		*median*	1.5	0.7
		*IQR*	4.0	2.2

**Table 3:** Overall compound motion (mm) during external (ER) and internal (IR) rotation. Abbreviations: IQR = interquartile range; SD = standard deviation

			Compound Motion (mm) during ER	Compound Motion (mm) during IR
**0°**	**Native**	*mean ± SD*	6.1 ± 4.8	6.1 ± 4.4
		*median*	4.7	4.3
		*IQR*	7.0	5.0
	**Elliptical**	*mean ± SD*	4.3 ± 2.6	5.7 ± 6.0
		*median*	4.1	2.5
		*IQR*	2.8	8.6
	**Sphere**	*mean ± SD*	3.8 ± 2.5	4.1 ± 4.0
		*median*	3.0	1.8
		*IQR*	2.8	7.4
**30°**	**Native**	*mean ± SD*	5.2 ± 3.2	5.3 ± 4.3
		*median*	5.6	3.6
		*IQR*	6.2	6.3
	**Elliptical**	*mean ± SD*	3.7 ± 2.0	4.6 ± 3.8
		*median*	3.8	3.1
		*IQR*	2.7	6.8
	**Sphere**	*mean ± SD*	3.7 ± 2.6	4.5 ± 3.7
		*median*	3.1	3.4
		*IQR*	3.6	5.7
**45°**	**Native**	*mean ± SD*	5.6 ± 3.3	5.1 ± 4.7
		*median*	5.5	4.2
		*IQR*	3.3	0.3
	**Elliptical**	*mean ± SD*	3.4 ± 2.8	5.5 ± 4.3
		*median*	2.3	4.3
		*IQR*	5.0	6.9
	**Sphere**	*mean ± SD*	3.3 ± 2.6	5.6 ± 4.6
		*median*	2.6	4.0
		*IQR*	4.1	8.6
**60°**	**Native**	*mean ± SD*	6.3 ± 3.1	6.5 ± 5.4
		*median*	6.6	3.7
		*IQR*	1.7	9.0
	**Elliptical**	*mean ± SD*	3.2 ± 2.8	6.8 ± 5.1
		*median*	2.4	5.0
		*IQR*	2.5	8.6
	**Sphere**	*mean ± SD*	3.8 ± 3.3	7.2 ± 5.7
		*median*	2.9	5.5
		*IQR*	3.6	10.3

### Radius of curvature

When compared to the native humeral head, only the spherical design allowed for restoration of the radius of curvature in the anteroposterior dimension (sphere: P = 0.05; elliptical: P = 0.01). However, none of the two implant designs achieved a similar radius of curvature in the superoinferior dimension when compared to the native humeral head (sphere: P = 0.03, elliptical: P = 0.02; Tables 4 and 5).

**Table 4:** Radius of curvature (mm) of the anteroposterior and superoinferior dimension. Abbreviations: IQR = interquartile range, SD = standard deviation

		Radius of Curvature (mm)	
		Anteroposterior Dimension	Superoinferior Dimension
**Native**	*mean ± SD*	26.6 ± 3.1	26.0 ± 3.2
	*median*	26.2	27.1
	*IQR*	4.9	1.8
**Elliptical**	*mean ± SD*	23.1 ± 1.7	24.6 ± 2.7
	*median*	22.5	24.6
	*IQR*	3.1	2.2
**Sphere**	*mean ± SD*	24.0 ± 2.7	24.7 ± 2.5
	*median*	24.1	24.1
	*IQR*	4.2	2.4

**Table 5:** Comparison of radius of curvature between the native humeral head and the two implant designs. * Indicates statistical significance. Abbreviations: CI = confidence interval

Dimension	Comparison	Contrast	95% CI	P-Value
**Anteroposterior**	Elliptical vs. Native	-3.45	-6.03 - -0.87	0.01*
	Sphere vs. Native	-2.60	-5.18 - -0.02	0.05
**Superoinferior**	Elliptical vs. Native	-1.36	-2.51 - -0.21	0.02*
	Sphere vs. Native	-1.31	-2.46 - -0.17	0.03*

## Discussion

The most important finding of the present study was that spherical and elliptical heads demonstrated similar obligate glenohumeral translation during axial rotation at all abduction angles following TSA. Although similar measurements of posterior, inferior, and compound translational motion for elliptical and spherical heads were generated during external rotation at each tested abduction angle, these implants yielded less posterior translation when compared to the native humeral head. These findings indicate that surgical implants may alter the mechanical environment of the shoulder joint, allowing for less translational movement of the humerus after TSA.

In the context of internal rotation at resting position, the spherical head design demonstrated more anterior translation than the elliptical head. Moreover, the spherical head yielded greater anterior translation and compound motion than both the elliptical and native humeral heads at 60° of abduction with internal rotation. These observations may be explained by the influence of the spherical implant shape, which has more material on the posterior side that may be involved in pushing the humeral head anteriorly during internal rotation. Further, the anterior capsule was surgically opened to perform the arthroplasty, while the posterior capsule was left untouched. This may have inadvertently altered the structure of the joint, allowing the humeral head to translate anteriorly during internal rotation to a greater degree after arthroplasty, independent of the implant shape. Consistently, Massimini et al. demonstrated that elongation of the SGHL, MGHL, and IGHL during shoulder rotational movement influenced the degree of glenohumeral translation [28]. Thus, changes in implant obligate translation may be a result of varying tightness of soft tissue restraints after arthroplasty, reflecting true surgical consequences of capsule and tendon reconstructions during TSA.

Previous biomechanical studies have shown that a non-spherical prosthetic head more accurately replicated native glenohumeral joint properties in terms of kinematics, translation, and rotational range of motion [12–14]. More importantly, the non-spherical humeral head design resulted in increased glenohumeral translation during humeral axial rotation when compared to the spherical head design [13, 15]. However, these studies were limited to the prior resection of rotator cuff muscles and capsuloligamentous structures, leaving the effect of these soft tissue restraints on glenohumeral translation unknown. Further, prior work did not prove that the translations occurring at the glenohumeral joint were obligate, as no oppositely directed force was applied [1, 13].

In contrast, the present study showed no statistically significant difference in obligate translation between elliptical and spherical heads during both external and internal rotation. This observation may be explained due to the spherical design containing more physical material in the anteroposterior dimension when compared to the elliptical head [7, 8]. As tightening of the anterior part (during external rotation) or posterior part (during internal rotation) of the capsule pushes the spherical head more posteriorly or anteriorly during axial rotation, this may counterbalance the generally greater translational motion of elliptical heads having been reported in models not considering the effect of soft tissue restraints [13]. Due to these characteristic features of its shape, the spherical design generally has greater magnitude of translation at higher abduction angles, with the glenohumeral joint being more constraint along with increased tightness of the capsule. Thus, forces from the rotator cuff muscles and the capsule pressures itself may induce greater amounts of translation motion either anteriorly or posteriorly. Consequently, this trend was especially noticed during internal rotation, as the posterior part of the capsule was left untouched while performing the TSA. Accordingly, the spherical design showed significantly more obligate anterior translation and compound motion at 60° of abduction during internal rotation when compared to the resting position. At lower abduction angles, however, the capsule is looser with less engagement with the rotator cuff muscles.

Anatomic studies have reported that the humeral head diverges to elliptical in the anterior–posterior dimension at the periphery of the articular margin, having roughly an 8–12% difference in head radius when comparing frontal and sagittal planes [10, 29]. With the development of glenohumeral osteoarthritis, initial pathologic deformations are commonly observed at the humeral side, which can cause further progression of this elliptical shape, especially in cases of severe disease [9, 30, 31]. However, neither the elliptical nor the spherical design allowed for replicating the native radius of curvature. Although Habermeyer et al. found that 82.2% of patients who underwent anatomic shoulder arthroplasty for treatment of primary osteoarthritis had an aspherical humeral head shape, this observation also implied that 17.8% of the evaluated humeral heads were still spherical in shape, bringing greater confusion as to which patient would benefit more from a spherical head design rather than from an elliptical one [9]. Thus, these inconsistencies in the anatomy of each individual specimen, with the humeral head being either more spherical or elliptical in shape and differing tightness of the glenohumeral joint capsule, may further contribute to the relatively large variations of differences between spherical and elliptical heads, without reaching significance in favor of one of the two head designs.

While increased translation may resemble native glenohumeral kinematics more accurately, [12, 13] this may also lead to more eccentric loading on and greater micromotion of the glenoid component in the setting of TSA. As excessive micromotion of the glenoid component has been suggested as a significant risk factor for glenoid loosening in the long-term, [5, 32–34] these biomechanical time-zero findings may be of clinical importance, alleviating concerns of implant loosening due to increased rocking motion.

There were several limitations to the study. Humeral head prosthetic design may show a different effect in vivo when compared to observations during laboratory cadaveric testing. Further, the inconsistencies in the anatomy of each individual specimen, with the humeral head either being more elliptical or spherical in shape, may have further influenced the results. In addition, specimens with moderate to severe osteoarthritis were excluded from the study, as varying degrees of osteoarthritic changes may have influenced translational measurements. Moreover, the shoulder model of the present study was not able to account for differing tightness of the glenohumeral joint capsule, which may have caused alterations in translational motion of the humeral head. Lastly, the anteroinferior capsule was opened during surgical replacement while preserving the anterior IGHL, to to accurately visualize the resection plane of the humeral head. As switching of prosthetic head shapes and sizes had to be frequently performed during testing, a capsular repair was infeasible, potentially increasing anterior and decreasing posterior translation during rotational testing.

## Conclusion

In the setting of TSA, elliptical and spherical head implants showed similar obligate translation and overall compound motion during axial rotation. A gained understanding of the consequences of implant head shape in TSA may guide future surgical implant choice for better recreation of native shoulder mechanics and potentially improved patient outcomes.

## Data Availability

The datasets used and/or analysed during the current study are available from the corresponding author on reasonable request.

## List of abbreviations

IGH: Inferior glenohumeral ligament
IQR: Interquartile range
MGHL: Middle glenohumeral ligament
PVC: Poly-vinyl chloride
SD: Standard deviation
SGHL: Superior glenohumeral ligament
TSA: Total shoulder arthroplasty
USA: United States of America
